# A comparative analysis of clinical quality and readability of neuropathic pain patient information leaflets: Türkiye versus the United States

**DOI:** 10.55730/1300-0144.6341

**Published:** 2026-04-28

**Authors:** Gönül SARI, Argun PİRE

**Affiliations:** 1 Division of Pain Medicine, Department of Anesthesiology and Reanimation, Faculty of Medicine, Aydın Adnan Menderes University, Aydın, Turkiye; 2 Division of Pain Medicine, Department of Anesthesiology and Reanimation, Faculty of Medicine, Ege University, İzmir, Turkiye

**Keywords:** Health literacy, neuropathic pain, patient information leaflets, readability

## Abstract

**Background/aim:**

Neuropathic pain management requires high patient adherence and complex dose-titration regimens, making the readability of patient information leaflets (PILs) a critical patient safety factor. This study aimed to compare the clinical content quality and linguistic readability of PILs for neuropathic pain medications between Türkiye (TİTCK) and the United States (DailyMed).

**Materials and methods:**

A total of 11 pairs of innovator medications were analyzed. Clinical content was assessed using the 15-item Keystone Clinical Content Checklist (KCCC). Readability was assessed using the Ateşman index for Turkish texts and the Flesch Reading Ease Score (FRES) for English texts. Information quality and reliability were evaluated using DISCERN, the Global Quality Score (GQS), and JAMA benchmarks.

**Results:**

TİTCK leaflets exhibited significantly higher word counts (2652.27 ± 591.6) and sentence counts (180.55 ± 30.7) than the US group (564.09 ± 75.71 and 28.18 ± 4.48, respectively; p < 0.001). While the Turkish group demonstrated superior clinical exhaustiveness (KCCC: 14.82 ± 0.40 versus 11.91 ± 1.22; p < 0.001), both groups remained within the “Difficult/Academic” readability category, with no significant difference between Ateşman and FRES scores (p = 0.4056). Notably, the US group demonstrated significantly higher educational utility (GQS: 4.27 ± 0.47 versus 3.27 ± 0.47; p = 0.0049), despite its lower content volume.

**Conclusion:**

The comparison of regulatory frameworks revealed a structural trade-off: while the TİTCK model offered superior clinical exhaustiveness, the FDA model provided significantly higher educational utility. Despite these differing priorities, neither model provided a readability advantage, as both remained within the “Difficult” category for the target patient population. These findings suggest that the high information density of the Turkish model acts as a significant barrier to effective health communication.

## 1. Introduction

Patient information leaflets (PILs) serve as fundamental educational tools that enable patients to manage their pharmacotherapy safely, accurately, and effectively. By presenting critical clinical data—including specific dosage regimens, administration instructions, potential adverse reactions, and contraindications—these documents aim to enhance patient awareness, reinforce treatment adherence, and maximize medication safety [1]. However, current literature suggests that the quality and functional comprehensibility of written medication information remain among the most persistent barriers to effective health communication and therapeutic success [2,3].

In clinical practice, particularly in the management of chronic and multisystemic conditions such as neuropathic pain, the therapeutic agents prescribed for this condition elevate this issue to a critical patient safety dimension. The intricate dose-titration protocols and potential adverse-effect profiles associated with these agents transform document clarity from a secondary concern into a fundamental component of patient safety [4]. As highlighted by Tang et al. (2025), dense textual structures and technical terminology act as primary cognitive barriers that prevent patients from transforming raw information into actionable knowledge [3]. These barriers pose a significantly greater risk for patients with neuropathic pain, whose cognitive processing is already strained by polypharmacy, advanced age, and the persistent “cognitive load” imposed by chronic pain [4,5]. Furthermore, linguistic complexity may trigger communication breakdowns not only among patients but also among healthcare professionals, who may misinterpret standard frequency terminology [6]. This situation creates an additional burden for physicians, who may spend limited counseling time clarifying confusing leaflets rather than providing patient education.

The quality of medication information is inextricably linked to both regulatory frameworks and corporate standards. Alzarea et al. (2025) noted that although multinational companies often provide higher-quality content than local firms, they frequently fail to achieve an optimal balance between “clinical exhaustiveness” and “user-friendliness” [7]. Simultaneously, the inadequacy of regulatory efforts aimed at simplifying patient materials remains a well-documented phenomenon in Europe [8]. Piñero-López et al. (2016) observed that despite the guidelines issued by the European Medicines Agency (EMA), no significant improvement in readability has been achieved; on the contrary, text volume has continued to increase [8,9].

At this juncture, two contrasting regulatory philosophies emerge within the global pharmaceutical ecosystem. The United States Food and Drug Administration (FDA) model recognizes health literacy as a dynamic systems-level issue. Since only 12% of US adults are proficient in health literacy, the Plain Writing Act of 2010 and the July 2022 FDA guidance function as system-level adaptations designed to accommodate these limitations rather than reflecting improvements in literacy levels. The implementation of plain-language guidelines serves as a strategic approach to improving the comprehensibility of complex health information and enhancing patient education, particularly for individuals with limited health literacy [10–12]. Conversely, regulations in Türkiye, which follow the directives of the Turkish Medicines and Medical Devices Agency (TİTCK) in alignment with EMA standards, are rooted in a philosophy of encyclopedic completeness [13,14]. While this model maximizes clinical content and legal protection, it carries the risk of confining documents to the “Difficult” readability category (Ateşman < 45) [5,15].

The gravity of the situation in Türkiye is underscored by the fact that 75% of the population possesses limited health literacy [16]. According to OECD 2025 data, the finding that 28% of even young adults have an educational attainment level below high school provides a plausible explanation for why TİTCK leaflets constitute a substantial accessibility barrier [17]. This structural gap may contribute to medication misuse, life-threatening adverse effects, and the failure to achieve the expected therapeutic benefits.

This study aimed to evaluate and compare the PILs of 11 innovator medications used in neuropathic pain management, retrieved from the TİTCK and DailyMed databases, in terms of readability, comprehensiveness, and quality. By analyzing structural differences, this study sought to contextualize the findings within the national health literacy landscapes of both countries.

## 2. Materials and methods

This study was designed as a cross-sectional, descriptive document analysis examining PILs for pharmacological agents used in neuropathic pain management from structural and linguistic perspectives. The study was based on the official databases of Türkiye (TİTCK) [13,14] and the United States (DailyMed) [10,11]. The systematic identification, screening, and inclusion process of the study sample was conducted in accordance with the PRISMA framework (Figure).

The sampling strategy was determined using a purposive sampling approach to construct a dataset that best reflects clinical practice while maintaining a high level of regulatory standardization. Medication selection was based on the following predefined criteria:

- Clinical guideline alignment: The agent must be listed as a first-line, second-line, or adjuvant treatment in current international neuropathic pain guidelines (IASP/NeuPSIG [18], NICE [19], EFNS [20]).
- Market synchronicity: The selected medications must be concurrently licensed and actively used in clinical practice in both countries (the US and Türkiye).
- Manufacturer standardization: To isolate regulatory differences as clearly as possible, the documents in both markets were required to be provided by the same innovator manufacturer.

In accordance with these criteria, 11 innovator medication pairs—Cipram (H. Lundbeck A/S, Copenhagen, Denmark), Cymbalta (Eli Lilly and Company, Indianapolis, IN, USA), Effexor XR (Pfizer Inc., New York, NY, USA), Lamictal (GlaxoSmithKline, Brentford, UK), Lyrica (Pfizer Inc., New York, NY, USA), Neurontin (Pfizer Inc., New York, NY, USA), Tegretol (Novartis Pharma AG, Basel, Switzerland), Topamax (Janssen Pharmaceuticals, Titusville, NJ, USA), Trileptal (Novartis Pharma AG, Basel, Switzerland), Vimpat (USB Pharma, Brussels, Belgium), and Wellbutrin XL (GlaxoSmithKline, Brentford, UK)—which constitute cornerstone therapies in neuropathic pain management, were included in the analysis. Generic medications were excluded to ensure that local marketing or manufacturing strategies did not overshadow the primary focus of the study: differences in corporate regulatory philosophies.

Data were retrieved in January 2026 from the TİTCK Drug Information Bank and the DailyMed database [11,14]. Current official master texts (PILs) representing all dosage forms available on the market were downloaded for each medication. Prior to analysis, the texts were digitally converted to plain-text format. To isolate patient-directed content, drug names, headings, manufacturer addresses, and legal disclaimers were removed, leaving only the patient-oriented informational sections for analysis [15,16].

The linguistic complexity of the texts was quantitatively assessed using fundamental parameters, including word, sentence, and syllable counts, as well as average word length [15,16]. To ensure standardization and minimize calculation errors, validated online tools were used for all analyses.

The Flesch Reading Ease Score (FRES), widely regarded as the gold standard for assessing the readability of English texts, was used [21]. The analysis was performed using the ReadabilityFormulas.com platform, a widely used readability assessment tool. The formula used for score calculation was as follows:


FRES=206.835-(1.015×ASL)-(84.6×ASW)

(ASL: average sentence length [words/sentence]; ASW: average syllables per word).

For the analysis of Turkish texts, the Ateşman index was preferred because it has been specifically validated for the agglutinative morphological structure of the Turkish language [22]. The analysis was conducted using the okunabilirlikindeksi.com interface, which digitally analyzes the linguistic parameters of Turkish texts. The index was based on the following formula:


R=198.825-(40.175×L_w)-(2.610×L_s)

(L_w: average syllables per word; L_s: average words per sentence).

Both indices generate scores ranging from 0 to 100. The difficulty categories of these scores and their corresponding estimated educational levels are presented in Table 1.

The clinical exhaustiveness and informational quality of the leaflets were evaluated using the following internationally recognized assessment tools:

- JAMA Benchmark score (0–4): Used to assess the transparency of the text based on four criteria: authorship, attribution, disclosure, and currency [23].
- DISCERN score (16–80): An internationally validated instrument designed to evaluate the quality and reliability of written consumer health information regarding treatment choices [24].
- Global quality score (GQS) (1–5): A scoring system used to evaluate the overall educational value, flow, and clinical utility of the leaflets. A score of 1 indicates poor quality and inadequate structural integrity, whereas a score of 5 represents excellent quality and high educational utility for patients [25].
- Keystone Clinical Content Checklist (KCCC): The KCCC was specifically developed for this study because no validated instrument was available to assess the clinical depth of patient information leaflets (Table 2). The checklist items were based on the mandatory clinical requirements defined in FDA regulation 21 CFR Part 201.57 and the European Union (EU) Directive 2001/83/EC [26,27]. Based on these regulatory frameworks, 15 essential clinical parameters were identified as necessary for patient safety. These 15 items were organized into four domains: clinical application (4 items), safety and risk warnings (6 items), error management (2 items), and logistics and identification (3 items). This structure was designed to ensure that the checklist evaluates both the presence of information and its practical utility in clinical settings. The content validity of the KCCC was evaluated by two independent expert panels. For the analysis, two blinded researchers independently scored each leaflet using a binary scoring system (1 = present; 0 = absent). Any scoring discrepancies were resolved through a formal consensus-building process, and interrater agreement was subsequently assessed statistically.

To ensure data reliability and minimize subjective bias, JAMA, DISCERN, GQS, and KCCC assessments were performed independently by two blinded researchers. Following the assessment process, the results were compared, and any discrepancies were resolved through consensus meetings until final agreement was achieved.

As this study was a cross-sectional document analysis based on publicly available official databases (TİTCK and DailyMed) and did not involve human participants, medical records, or animal subjects, institutional ethical approval was not required. All data were accessed in accordance with the open-access policies of the respective official institutions.

### 2.1. Statistical analysis

Statistical analyses were performed using SPSS version 28.0 (IBM Corp., Armonk, NY, USA). The normality of continuous variables was assessed using the Shapiro–Wilk test (given the sample size of n < 50) and visual inspection of histograms and Q–Q plots. Descriptive statistics were expressed as mean ± standard deviation (SD) for normally distributed variables and as median (minimum–maximum) values for nonnormally distributed data. Categorical variables were presented as frequencies and percentages.

Since the study employed a paired design (evaluating the same innovator medications across two different regulatory databases), comparative analyses were conducted using the paired-samples t-test for parametric data and the Wilcoxon signed-rank test for nonparametric distributions. The mean differences between groups were reported along with 95% confidence intervals (CIs) to indicate the magnitude and direction of the observed effects.

Interrater aggreement between the two independent researchers for the JAMA, DISCERN, GQS, and KCCC scores was intended to be quantified using the intraclass correlation coefficient (two-way mixed-effects model, absolute agreement) for continuous or ordinal data and Cohen’s kappa coefficient for categorical parameters. Statistical significance for all analyses was set at a two-tailed p < 0.05.

## 3. Results

Data regarding the structural and linguistic metrics of patient information leaflets for neuropathic pain medications from the United States (n = 11) and Türkiye (n = 11) are presented in Table 3. Statistical analyses revealed that the mean word count of the Turkish leaflets (2652.27 ± 591.6) was significantly higher than that of the US group (564.09 ± 75.71), indicating substantially greater textual volume (p < 0.001; 95% CI [1692, 2484]). Similarly, a significant difference was observed between the mean sentence counts of the Turkish group (180.55 ± 30.7) and the US group (28.18 ± 4.48), highlighting divergent approaches to information density (p < 0.001; 95% CI [131, 172]).

In terms of total syllable count, Turkish texts (7845.36 ± 1700.6) demonstrated substantially higher values than US texts (986.18 ± 143.76), further underscoring the structural complexity of the documentation (p < 0.001; 95% CI [5715, 8003]). The mean number of polysyllabic words (four or more syllables), an indicator of lexical complexity, was 758.64 ± 248.4 in Turkish leaflets compared with 80.73 ± 15.57 in US leaflets (p < 0.001; 95% CI [513, 842]).

Regarding average word length (L), the Turkish group (2.96 ± 0.04 syllables) exhibited significantly higher values than the US group (1.75 ± 0.08 syllables), reflecting the more complex morphological structure of Turkish medical terminology (p < 0.001; 95% CI [1.16, 1.27]). Conversely, the average sentence length (S) was greater in the US group (20.07 ± 2.44 words) than in the Turkish group (14.67 ± 1.8 words), representing a statistically significant difference (p < 0.001; 95% CI [−7.49, −3.32]).

Regarding readability levels, the mean Flesch Reading Ease Score (FRES) for the US group was 38.81 ± 7.33, whereas the mean Ateşman index score for the Turkish group was 41.17 ± 4.36. No statistically significant difference was observed between the two groups (p = 0.4056; 95% CI [−3.68, 8.39]), indicating that PILs in both countries remained within the “Difficult/Academic” readability category (Table 4).

In terms of information quality and clinical exhaustiveness, the Turkish group demonstrated a KCCC score of 14.82 ± 0.40, which was significantly higher than the US group’s score of 11.91 ± 1.22. This 2.91-unit difference was statistically significant (p < 0.001; 95%CI [1.99, 3.83]). Health information quality, as measured by DISCERN scores, was 66.45 ± 3.64 for the Turkish group and 62.18 ± 2.18 for the US group, reflecting a statistically significant difference in favor of the Turkish leaflets (p = 0.0148; 95% CI [1.04, 7.51]).

Reliability metrics, represented by JAMA scores, were identical between the two groups (3.00 ± 0.00), with no statistically significant difference observed (p = 1.0000). Notably, the Global Quality Score (GQS), which assesses educational utility, was 4.27 ± 0.47 for the US group and 3.27 ± 0.47 for the Turkish group. This difference was statistically significant (p = 0.0049; 95% CI [−1.42, −0.57]), suggesting that despite lower content volume, the US model provides higher perceived educational quality for patients.

Data concerning the interrater agreement analysis are presented in Table 5. In both the US and Turkish samples, complete agreement (100%, n = 11) was achieved for KCCC and JAMA scoring, supporting the consistency of these assessment metrics. Regarding DISCERN scores, the US group demonstrated a 90.9% agreement rate (n = 10) with a 9.1% discrepancy rate (n = 1), whereas the Turkish group demonstrated an 81.8% agreement rate (n = 9) with an 18.2% discrepancy rate (n = 2). In the GQS assessments, the US group demonstrated complete agreement (100%, n = 11), whereas the Turkish group demonstrated a 90.9% agreement rate (n = 10) with a 9.1% discrepancy rate (n = 1). These high levels of agreement between independent evaluators suggest that the findings are reproducible and minimally affected by individual interpretative bias.

## 4. Discussion

The primary finding of this study is that medications containing identical active ingredients exhibit significant disparities in both informational content and readability when governed by different regulatory authorities. Leaflets retrieved from the TİTCK database demonstrated higher KCCC scores than those retrieved from DailyMed. However, this level of clinical exhaustiveness introduces a substantial barrier to linguistic accessibility. The fact that TİTCK-sourced documents contain approximately four times the textual volume of their US counterparts imposes a substantial cognitive burden on patients.

Despite the superior clinical completeness of TİTCK leaflets, their lower GQS scores reflect an inverse relationship between information volume and patient accessibility [7]. Regulatory models that prioritize encyclopedic detail may create dense textual barriers that paradoxically hinder access to actionable knowledge. This level of information volume may be particularly overwhelming for patients with neuropathic pain, who already experience substantial physical and cognitive burdens. Ultimately, patient safety is more likely to be enhanced by clear, actionable information than by excessive textual volume [1,28].

The absence of a significant difference between US FRES scores and Turkish Ateşman scores underscores a broader systemic challenge in medical documentation. Across all analyzed documents, the recommended eighth-grade reading level for patient education remained largely unmet [28]. Despite the Plain Writing Act of 2010 and the 2022 FDA guidelines, academic jargon continues to dominate US leaflets [10,11]. Although educational attainment levels in the US are relatively high, PIAAC data indicate that many adults continue to struggle with processing complex medical information. This reality may help explain the FDA’s strategy of prioritizing simplified, action-oriented instructions over technical depth. Maintaining materials within the “Difficult” readability category may place patients in a more passive role during treatment processes [29,30]. Furthermore, research suggests that even generative artificial intelligence (AI) systems struggle to overcome this “Difficult” readability barrier, indicating that medical language itself may remain fundamentally inaccessible to many patients [31].

The differing missions of national health authorities fundamentally shape the structure of PILs. The TİTCK model, following EU Directive 2001/83/EC and EMA guidelines, adopts a philosophy of encyclopedic completeness that views patients as stakeholders entitled to extensive medical detail [13,14]. However, this approach results in substantial textual expansion—a phenomenon also reported in the European model—that may function more as a legal safeguard for manufacturers than as a practical guide for patients [8,9]. Conversely, the US model prioritizes action-oriented simplification under the Plain Writing Act of 2010 [10]. While this approach improves accessibility, lower KCCC scores suggest that technical depth may sometimes be sacrificed for brevity. Given that 75% of Turkish adults have limited health literacy (OECD, 2025), the dense volume of TİTCK leaflets may pose a substantial risk of misunderstanding [16,17]. Since technical jargon may hinder comprehension even among experts, this lack of clarity may render these documents functionally inaccessible for patients with limited cognitive reserves [6].

Patients with neuropathic pain inherently experience cognitive limitations due to pain-related fatigue and the sedative effects of medications such as gabapentinoids. Previous studies have demonstrated a significant negative correlation between health literacy and pain severity [32], as higher health literacy levels are associated with improved self-management [30]. In this context, the 2652-word volume and “Difficult” readability category (Ateşman < 45) of TİTCK leaflets may contribute to cognitive overload by exceeding working memory capacity [33]. Since successful pain management depends on strict adherence to complex dose-titration regimens, dense and incomprehensible texts may contribute to treatment discontinuation rather than patient protection. Therefore, improving readability may represent an important patient safety strategy for enhancing treatment adherence and reducing neuropathic pain-related morbidity [32,34].

Text simplification alone may not ensure safe medication use; for populations with limited literacy or reduced cognitive reserves, nontextual aids such as pictograms may be essential for enhancing comprehension [35]. The “Difficult” readability profile of TİTCK leaflets may negatively affect patient activation, as dense information may place patients in a more passive role during treatment processes [36]. Current evidence supports transitioning from static paper documents to dynamic digital education models that provide information in manageable segments, which may increase treatment adherence by up to 82% [33,37]. While generative AI offers potential for personalization, its current outputs continue to exhibit high linguistic complexity, thereby limiting its effectiveness in patient communication [5,31]. Beyond these clinical implications, the literature indicates that the inability to understand PILs may increase emergency department visits related to medication misuse and may impose an annual economic burden exceeding USD 42 billion on the global healthcare system because of medication waste associated with nonadherence [34,38,39].

In conclusion, this study suggests that clinical depth does not necessarily guarantee linguistic readability in neuropathic pain leaflets. While TİTCK-sourced documents provide near-complete clinical exhaustiveness, their “Difficult” readability category may pose a substantial cognitive barrier, particularly given the high prevalence of limited health literacy in Türkiye. Conversely, the FDA model appears to achieve higher accessibility at the expense of some technical depth.

To bridge this gap, regulatory authorities should consider moving away from static, dense documentation and adopting simplified formats targeting an eighth-grade reading level. Leveraging technological advancements, particularly AI-supported dynamic education models, may be valuable for delivering personalized and manageable information. Ultimately, successful neuropathic pain management may depend on establishing a health communication ecosystem that is accessible, comprehensible, and aligned with patients’ cognitive capacities.

### 4.1. Limitations

This study is limited to 11 innovator medication pairs that represent commonly used therapies in neuropathic pain management. The readability indices employed, such as the Ateşman index and FRES, primarily focus on mathematical parameters, including word length and sentence structure. Although comparing scores derived from different linguistic systems (English and Turkish) represents a methodological limitation because of structural language differences, both indices use a comparable 0–100 scale calibrated for reading difficulty. Consequently, these formulas cannot fully evaluate elements related to cognitive ergonomics, such as visual hierarchy, font size, line spacing, or the effectiveness of pictogram support.

This study is limited to 11 innovator medication pairs. Although this purposive sampling strategy allows for a standardized comparison of regulatory differences, the relatively small sample size limits the generalizability of the findings. Furthermore, although the findings objectively demonstrate the structural difficulty of the texts, this study did not directly evaluate actual patient comprehension levels or medication administration success through field-based assessments involving individuals from diverse sociocultural backgrounds in real-world clinical settings.

## Figures and Tables

**Figure:**
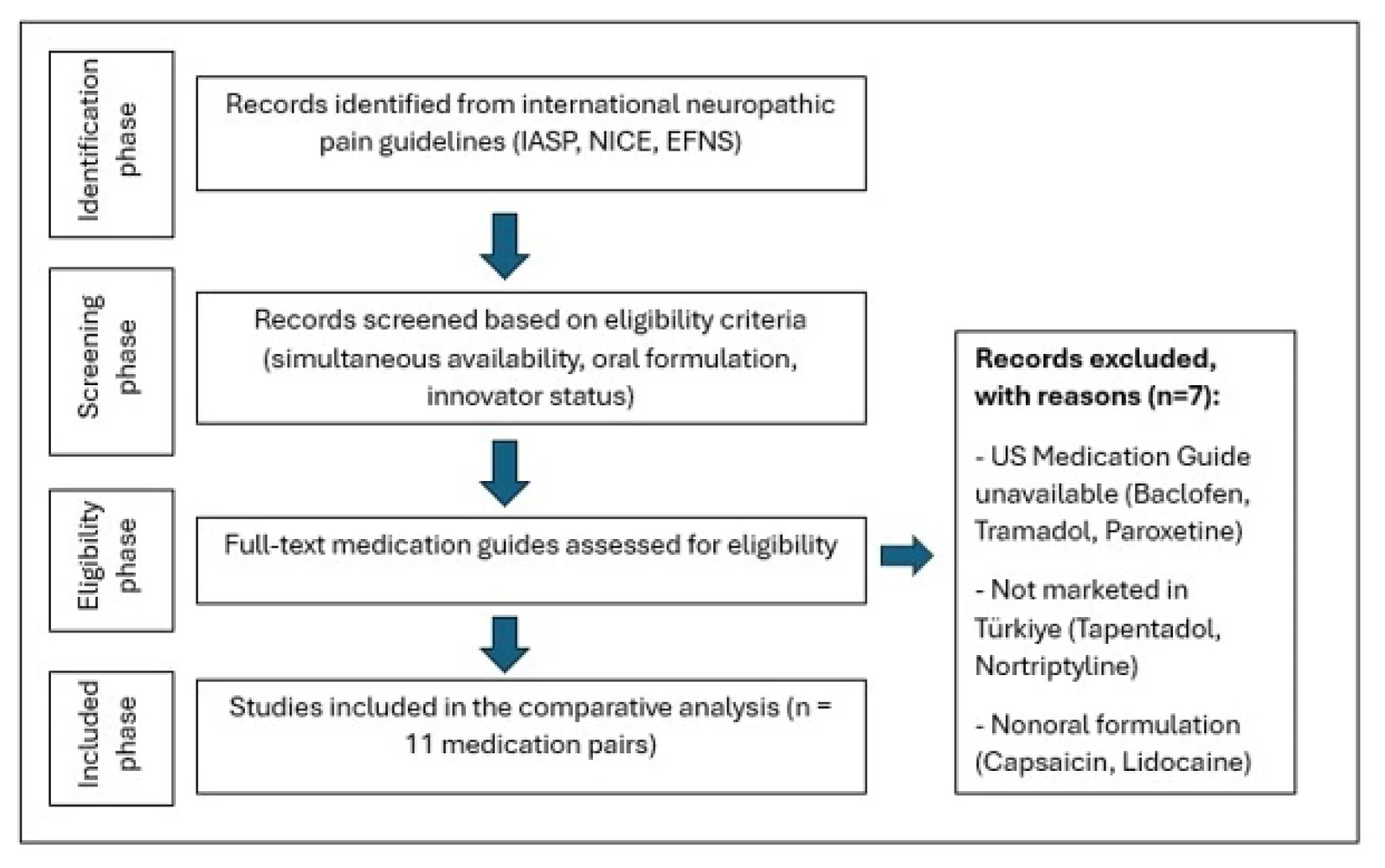
PRISMA flow diagram of the sample inclusion process.

**Table 1:** Comparative scoring and interpretation of the FRES (English) and Ateşman (Turkish) readability indices.

Index	Score range	Difficulty level	Estimated educational level
Flesch Reading Ease (FRES) (English)	90–100	Very easy	5th grade (primary school)
	80–89	Easy	6th grade
	70–79	Fairly easy	7th grade
	60–69	Standard/plain	8th–9th grade (middle school)
	50–59	Fairly difficult	10th–12th Grade (high school)
	30–49	Difficult	College/university
	0–29	Very difficult	Graduate/professional
Ateşman Readability Index (Turkish)	90–100	Very easy	Grade 1–5 (primary school)
	70–89	Easy	Grade 6–8 (middle school)
	50–69	Medium	Grade 9–12 (high school)
	30–49	Difficult	University/academic
	1–29	Very difficult	Postgraduate/professional

**Table 2:** The 15-item Keystone Clinical Content Checklist (KCCC) for assessing the clinical quality and exhaustiveness of patient information leaflets.

Domain	Item no	Assessment question
Clinical application	1	Does the leaflet specify the therapeutic indications for the medication?
	2	Is the recommended dosage clearly described?
	3	Is the method of administration (how to take the medication) clearly stated?
	4	Is the expected duration of treatment mentioned?
Safety and risk Warnings	5	Are the contraindications for the medication listed?
	6	Are the common side effects reported?
	7	Are serious side effects and symptoms requiring urgent medical attention highlighted?
	8	Are potential drug–drug interactions explained?
	9	Are specific instructions provided for special populations (e.g., pregnancy, elderly)?
	10	Is there a warning regarding the effects on driving or using machinery?
Error management	11	Are instructions provided on what to do if a dose is missed?
	12	Is there guidance on how to manage an overdose?
Logistics and identification	13	Are the storage conditions clearly stated?
	14	Are disposal instructions for unused medication provided?
	15	Is there a physical description of the medication (color, shape, markings)?

Table note: The checklist consists of 15 essential items categorized into four primary domains: Clinical application, Safety and risk warnings, Error management, and Logistics and identification. Each item was evaluated using a binary scoring system in which a score of 1 was assigned when the information was present and a score of 0 when the information was absent or insufficient. The total KCCC score (ranging from 0 to 15) serves as an objective measure of the clinical exhaustiveness and informational depth provided to patients.

**Table 3:** Comparative analysis of the structural and linguistic characteristics of neuropathic pain patient information leaflets.

Drug name	Word count (US/Türkiye)	Sentence count (US/Türkiye)	Syllable count (US/Türkiye)	Polysyllabic words (4+) (US/Türkiye)	Mean word length (L) (US/Türkiye)	Mean sentence length (S) (US/Türkiye)
Wellbutrin	569/3469	28/202	970/10182	78/1048	1.70/2.93	20.32/17.17
Vimpat	501/2432	30/168	811/7126	62/642	1.62/2.93	16.70/14.47
Trileptal	436/2843	23/198	765/8279	68/923	1.75/2.90	18.96/14.43
Topamax	557/3582	28/188	969/10479	81/1147	1.74/2.93	19.89/19.05
Neurontin	509/2697	24/208	881/8012	74/798	1.73/2.97	21.21/12.96
Lyrica	708/2684	30/198	1232/7918	106/792	1.74/2.95	23.60/13.55
Lamictal	629/2642	28/178	1056/8058	85/742	1.68/3.05	22.46/14.84
Effexor	577/2711	29/194	1087/8018	92/785	1.88/2.95	19.90/14.01
Cymbalta	643/2486	38/172	1179/7383	102/762	1.83/2.97	16.92/14.45
Cipram	514/1342	22/96	952/4052	84/422	1.85/3.02	23.36/13.97
Tegretol	562/2287	32/184	946/6792	56/284	1.68/2.97	17.56/12.43
Mean (SD)	564/2652	28/180	986/7845	81/758	1.74/2.96	20.07/14.67
95% CI (Difference)	[1692, 2484]	[131, 172]	[5715, 8003]	[513, 842]	[1.16, 1.27]	[−7.49, −3.32]
p value	<0.001	<0.001	<0.001	<0.001	<0.001	<0.001

Table note: The bottom rows summarize the overall comparison of group averages (mean ± SD) and p values between the two countries, while the 95% confidence interval (CI) represents the mean difference between the groups. Statistical significance values were calculated using the Wilcoxon signed-rank test for nonnormally distributed data and the paired-samples t-test for normally distributed data.

**Table 4:** Statistical comparison of readability (FRES/Ateşman), information quality, and reliability scores between the US and Turkish groups.

Metric	US Mean ± SD	Türkiye mean ± SD	95% CI	p-value
Readability (FRES/Ateşman)	38.81 (7.33)	41.17 (4.36)	[−3.68, 8.39]	0.4056
KCCC (Clinical exhaustiveness)	11.91 (1.22)	14.81 (0.40)	[1.98, 3.83]	<0.001*
JAMA (Reliability assessment)	3.00 (0.00)	3.00 (0.00)	-	1.000
DISCERN (Information quality)	62.18 (2.18)	66.45 (3.64)	[1.03, 7.50]	0.0147*
GQS (Educational Utility)	4.27 (0.47)	3.27 (0.47)	[−1.42, -0.57]	0.0049*

Table note: Data are presented as mean (standard deviation). The Flesch Reading Ease Score (FRES) was used for the US group, whereas the Ateşman readability index was used for the Türkiye group.

Abbreviations: KCCC, Keystone Clinical Content Checklist; JAMA, Journal of the American Medical Association benchmarks; DISCERN, quality scale for health information; GQS, Global Quality Score. The 95% CI represents the confidence interval for the difference between the two groups. Statistical significance values were calculated using the paired-samples t-test for normally distributed variables and the Wilcoxon signed-rank test for nonnormally distributed data.

* p < 0.05 indicates statistical significance.

**Table 5:** Comparison of interrater agreement and reliability measures for quality, reliability, and clinical content scores.

Test	Country	Agreement (n, %)	Difference (n, %)
KCCC	US	11 (100%)	0 (0%)
	Türkiye	11 (100%)	0 (0%)
JAMA	US	11 (100%)	0 (0%)
	Türkiye	11 (100%)	0 (0%)
DISCERN	US	10 (90.9%)	1 (9.1%)
	Türkiye	9 (81.8%)	2 (18.2%)
GQS	US	11 (100%)	0 (0%)
	Türkiye	10 (90.9%)	1 (9.1%)

Table note: Data are presented as frequencies (n) and percentages (%). “Agreement” indicates that both independent raters assigned the same score, whereas “Difference” indicates a scoring discrepancy between the raters.

Abbreviations: KCCC, Keystone Clinical Content Checklist; JAMA, Journal of the American Medical Association benchmarks; DISCERN, quality scale for health information; GQS, Global Quality Score.

## Data Availability

The datasets (patient information leaflets) analyzed during the current study are available in the official repositories of TİTCK (https://www.titck.gov.tr) and DailyMed (https://dailymed.nlm.nih.gov).
